# Prevalence of Intestinal Parasitic Infestations Among School Going Children of Shree Krishna Sanskrit Tatha Sadharan Madhaymik Vidhyalaya, Surkhet: An Observational Study

**DOI:** 10.31729/jnma.9105

**Published:** 2025-07-01

**Authors:** Damber Khadka, Sumana lama, Bikash Khadka

**Affiliations:** 1 Province Hospital Surkhet, Birendranagar, Surkhet, Nepal; 2 Shree Birendra Hospital, Chhauni, Kathmandu, Nepal

**Keywords:** *worm infestation*, *parasitosis*, *prevalence*, *school children*

## Abstract

**Introduction:**

Parasitosis is a major public health concern in developing countries, significantly contributing to childhood malnutrition, anemia, stunted physical and mental growth, and social problems. This study aimed to determine the prevalence of intestinal worm infestation and its associated risk factors among school going children.

**Methods:**

A descriptive study was done among school - going children of Shree Krishna Sanskrit Tatha Sadharan Madhaymik Vidhyalaya from March 20 to May 31, 2025. A purposive (non-probability) sampling technique was employed. Prior Informed consent was obtained from participants and guardians. Data were collected using a structured, self-designed proforma. Data analysis was done using SPSS 20.

**Results:**

The prevalence of intestinal parasitic infestation among school-going children in this study was 129 (52.41%; 95.00% CI: 46.00%-58.82%). Among children aged 6-9 years, the prevalence was 47 (82.45%), and among females, it was 75 (55.55%). Infestation was found in 46 (92.00%) of children who washed hands with water only after defecation and in 53 (77.94%) of those who had not taken anthelmintic medication.

**Conclusions:**

Parasitic infestations in children included in this study was higher as compared to other published study in Nepal.

## INTRODUCTION

Intestinal parasitic infestation is one of the major public health concerns and are leading cause of illness and death in developing countries, including Nepal.^1^ Studies have shown a higher incidence of worm infestation among school children.^23^ The widespread distribution and serious global impact of intestinal parasitic infestation are underscored by the inclusion of Soil-transmitted helminths infections as part of the World Health Organization's (WHO) Neglected Tropical Diseases (NTDs) initiative.^4^ It is estimated that approximately 3.5 billion people worldwide are affected, with around 450 million experiencing illness due to these infections most of whom were children. ^5^ This vulnerability is often linked to improper sanitation and poor hygiene practices among young children.^6^ In addition, parasitic infestation can lead to several adverse outcomes such as childhood malnutrition, anemia, stunted physical, and mental growth, psychosocial problems.^7^

Several studies have dealt with the prevalence and risk factors associated with intestinal parasitic infestations and have mostly concluded that the spectrum of intestinal parasitic infestations differs across regions, and poor hygiene and low socioeconomic conditions along with inadequate medical facilities and lack of safe drinking water are the common risk factors.^6,8^ Furthermore, in the context of Surkhet, little is known about prevalence and its risk factors of intestinal parasitic infections. To fill this gap, a descriptive study was conducted to determine the prevalence of intestinal worm infestation and its associated risk factors among school going children.

## METHODS

An observational cross-section study was conducted in, Shree Krishna Sanskrit Tatha Sadharan Mahyamik. Vidyalaya, Surkhet from 20th March 2025 to 31st May 2025. Shree Krishna Sanskrit Tatha Sadharan Ma. Vi., Surkhet is a public academic institute located in Birendranagar, Itram, Surkhet, Karnali Province, Nepal. The school is located in the capital of the Karnali province therefore, most of the students are from the remote districts of the Karnali province. There are 1100 students enrolled in class 1 to 10.

Ethical approval was obtained from Ethical Review Board of Nepal Health Research Council (Reference Number: 2166). The researcher visited the school and performed the general check-up of the selected participants only after getting informed consent from parents and assent from the school children on the presence of school administration or parents. Then the researcher administered the questionnaire and monitored the completeness of the questionnaire. The study included students from class 1 to 10 who submitted stool samples. Students below 6 years of age; those absent on the day of sample collection; and who did not provide stool samples were excluded. Convenient sampling technique was done for the selection of the study subjects. The sample size calculation was performed using;

n = Z^2^ × p × q/e^2^

= 1.962 × 0.225 × (1-0.225)/0.05^2^

= 268

Where,

n = minimum required sample size
Z = 1.96 at 95% Confidence Interval (CI)
p = prevalenc=22.5%^9^
q = 1-p
e = margin of error = 5%

A semi structured interview proforma was devised to obtain the information on socio-demographic characteristics and hygienic behavior practices of the students. Sample collection container was distributed to students on the same day of data collection. Each day 20-30 students submitted stool samples, which were examined on the same day at the laboratory of the Province Hospital Surkhet. Statistical analysis was done using the IBM SPSS Statistics for Windows, version 20 (IBM Corp., Armonk, N.Y., USA).

## RESULTS

In this study the total number of stool sample collected were 246. The prevalence of intestinal parasitic infestation amongst school going children in this study was 129 (52.41%; 95% CI: 46.00%-58.82%), Among the positive samples, giardia lamblia infestation was 45 (18.29%) and ascaris lumbricoides 30 (12.19%), 28 (11.38%) samples showed mixed parasites infections (Table 1).

**Table 1:** Intestinal Parasitic Infestations Among School Going Children of Shree Krishna Sanskrit Tatha Sadharan Madhaymik Vidhyalaya, Surkhet (n = 246).

Intestinal Parasites	Positive case n(%)
Giardia lamblia	45(18.29)
Ascaris lumbricoides	30(12.19)
Mixed parasites	28(11.38)
Trichuris Trichura	12(4.87)
Hook worm	8(3.25)
Hymenolepes nana	6(2.43)
**Total**	**129(52.41)**

In sub-group analysis, the intestinal parasite was observed in 47 (82.45%) of children between age 6-9 years, 75 (55.55%) female, 45(57.69%) of children from class one to three and 99 (55.30%) mothers (Table 2).

**Table 2:** Parasitic Infestation as per age, gender, enrolled class and education of mother among school going children of Shree Krishna Sanskrit Tatha Sadharan Madhaymik Vidhyalaya, Surkhet (n= 246).

Variables	Parasite		Total
	Positive n(%)	Negative n(%)	
Age group(years)			
6-9 years	47(82.45)	10(17.55)	57
10-13 years	80(54.42)	67(45.58)	147
14-17 years	2(4.76)	40(95.24)	42
Gender			
Male	54(48.64)	57(51.36)	111
Female	75(55.55)	60(44.45)	135
Enrolled Class			
1-3	45(57.69)	33(42.31)	78
4-7	81(52.94)	72(47.06)	153
8-10	3(20.00)	12(80.00)	15
Mother education			
Illiterate	99(55.30)	80(44.70)	179
Below SLC	27(45.00)	33(55.00)	60
SLC and above	3(42.85)	4(57.15)	7

Upon analysis of potential risk factors, 53 (77.94%) children who did not take anthelmintic medication had parasitic infestation. Similarly, 109 (53.69%) children who drank tap water at home, 86(51.19%) with no water purification at home, and 46 (92.00%) who washed hands with water only after defecation had parasitic infestation. Among children with the habit of thumb sucking, 13 (52.00%) were affected. Additionally, 83 (56.46%) children who did not bath regularly and 116 (53.95%) who wore sandals also showed signs of parasitic infestation (Table 3).

**Table 3:** Risk factors related to worm infestation among school-going children of Shree Krishna Sanskrit Tatha Sadharan Madhyamik Vidyalaya, Surkhet (n=246).

Variables	Parasite		Total
	Positive n(%)	Negative n(%)	
Anti-helminthic consumption duration			
No	53(77.94)	15(22.06)	68
>3months	49(39.83)	74(60.17)	123
>6months	26(54.16)	22(45.84)	48
>1 years	1(14.28)	6(85.72)	7
Source of water at home			
Tap	109(53.69)	94(46.31)	203
Tank	6(66.66)	3(33.34)	9
Well	14(41.17)	20(58.83)	34
Water purification			
Yes	43(55.12)	35(44.88)	78
No	86(51.19)	82(48.81)	168
Handwash after defecation			
Soap and water	75(41.21)	107(58.79)	182
Ash water	8(57.14)	6(42.86)	14
Water only	46(92.00)	4(8.00)	50
Habit of nail biting			
Yes	58(55.23)	47(44.77)	105
No	71(50.35)	70(49.65)	141
Habit of thumb sucking			
Yes	13(52.00)	12(48.00)	25
No	116(52.48)	105(47.52)	221
Bathing			
Regular	46 (46.46)	53(53.54)	99
Irregular	83(56.46)	64(43.54)	147
Sandal wear			
Yes	116(53.95)	99(46.05)	215
No	13(41.94)	18(58.06)	31

## DISCUSSION

The overall prevalence of parasitic infestation among school going children in this study was 52.41% (129 out of 246), a rate notably higher than those reported in previous studies, which found prevalence rates of 26.31%, 27.00% & 27.62% respectively. ^8,10-12^ These differences might be due to contamination of drinking water supply and poor sanitation practice in the study sites.^13^ Moreover, the differences in geographical, climatic condition, variation in economic status, individual behavioural habit of selected children.^14-19^ Furthermore, we cannot deny the fact of open defecation.

The study showed that the prevalence of Giardia lamblia was higher than that of Ascaris Lumbricoides. This finding is consistence with the finding of some studies that showed higher prevalence i.e. 58.60%, 34.00% respectively.^14,15^ Those studies were conducted in rural area of Kathmandu and Kaski district among school- going children. Globally, G. lamblia is strongly linked to inadequate sanitation and identified as a primary cause of giardiasis, a diarrheal disease. Moreover, G. lamblia transmission typically occurs through contaminated food, water and soil and making it a leading cause for water borne illness among healthy individual.^16^ We observed a lower rate of helminth infections compared to protozoal infections in our study. The reduced prevalence may be partly due to routine deworming programs that provide antihelminthic medications to school-aged children. A meta-analysis from Nepal indicated that the burden of helminth infections tends to be higher in rural areas.^17^

The contributors of positive stool parasite were, children aged 6-9 years in grades 1 to 3 however, Dahal et al., observed higher rates among children over 15 years.^18^ This could be due to the fact that primary school children usually prefer outdoor games and they lack the knowledge regarding parasitic infection, hygiene and sanitation practices.^19^

Among the positive 129 samples, female had higher prevalence rate of parasitic infection (55.55%) than male. The study was in support with the study done by Dahal et al., in Kathmandu (female = 70.83%).^18^ The finding is contradictory with the study conducted by Shrestha et al. in city of Dharan. ^6^ This suggests that gender may or may not influence the occurrence of parasitic infections, depending on regional, environmental, and behavioural factors. Generally, higher mobility among males increases their exposure and risk of infection, while females may be at greater risk due to frequent soil contact while growing vegetables and consuming raw vegetables along with prepared meals more often than males.^20^ In rural areas, among children, girls are involved mostly in agricultural work that enhances exposure to intestinal parasitic infestation.^21^

Similarly, Mother education another contributor for parasitic infestation accounting for 55.30% mothers who were illiterate. Studies in India and Turkey that showed higher infection rates among children whose mother were uneducated.^22,23^

The finding of the study showed the highest prevalence among the children who didn't consume anti-helminthic medication (77.94%). Similar to the studies conducted in Kathmandu and Dharan.^6,18^ These findings highlight the potential effectiveness of routine anti-helminthic drug administration in children. Individuals who are aware of health and hygiene practices tend to ensure their children receive biannual anti-helminthic treatment. In contrast, underprivileged communities often neglect such measures and show little interest in participating in deworming programs, making their children more vulnerable to intestinal parasitic infections.^6^

In this study, the highest rate of parasitic infestation was observed among children who consumed water from taps. The finding was similar to the study conducted by Dahal et al., (tap water = 45.83%).^18^ The increased infection rate among children using direct tap water could be attributed to sewage contamination in the pipelines, likely caused by leaks that allow parasites to enter and spread through the water supply.^18^

Good personal hygiene practice is essential for preventing various disease. This study showed that the children washing hand with soap and water after defecation had parasitic infestation (41.21%) rate comparison to those with washing hand only using water (92.00%). This is similar to the studies conducted in India and Nepal that showed lower prevalence of parasitic infection among children practicing proper hand hygiene.^20, 22, 24,^ Children playing in outdoor environments are exposed to parasites, and failing to wash their hands before meals increase the risk of these parasites entering their bodies.^18,20^

Parasitic infestation among children with habit of nail biting was higher (55.23%) than not having the habit of nail biting. However, the finding was contradictory to the study conducted by sah et al. which showed no evidence of nail biting in prevalence of worm infestation.^20^ The possible reason may be due to most of the students belong to the primary class with age 6-9 years (Table 2) and may have lack of awareness regarding nail trimming and proper hand hygiene practices.

The prevalence was high (53.95%) among those children who wore sandals. This contrasts with the findings of a study conducted in the eastern region of Nepal, which could be attributed to the possibility that participants in our study may not have regularly worn effective footwear.^20^

Firstly, the study relied on single stool sample examination for detection of parasitic infections, that may have led to an under-estimation of the true prevalence. Secondly, the study shows a prevalence of single school in Surkhet and does not represent all school going children of Surkhet. Moreover, this is a prevalence study therefore, relationship between various sociodemographic variables and risk factors cannot be established from this study. Therefore, a study design sufficiently powered enough to test the factors associated with intestinal parasite infestation can be considered for future.

## CONCLUSIONS

The prevalence of intestinal parasitic infestations among school going children of Shree Krishna Sanskrit Tatha Sadharan Madhaymik Vidhyalaya, Surkhet was high in comparision to other studies. The important contributing factors were, mother's education, poor hygiene and sanitation practices.
